# Ole-Oxy, a Semi-Synthetic Analog of Oleuropein, Ameliorates Acute Skin and Colon Inflammation in Mice

**DOI:** 10.3390/antiox13111422

**Published:** 2024-11-20

**Authors:** Nikolaos V. Angelis, Efthymios Paronis, Georgia Sarikaki, Antonios Kyriakopoulos, Anna Agapaki, Pigi-Maria Niotopoulou, Christina C. Knai, Pavlos Alexakos, Odyssefs Liagkas, Konstantinos F. Mavreas, Constantin N. Baxevanis, Alexios-Leandros Skaltsounis, Ourania E. Tsitsilonis, Ioannis K. Kostakis

**Affiliations:** 1 Flow Cytometry Unit, Section of Animal and Human Physiology, Department of Biology, National and Kapodistrian University of Athens, Panepistimiopolis, Ilisia, 15784 Athens, Greece; nangelis@biol.uoa.gr (N.V.A.); eparonis@med.uoa.gr (E.P.); pniotopoulou@student.ethz.ch (P.-M.N.); cknais@med.uoa.gr (C.C.K.); o.liagkas@nyu.edu (O.L.); cnbaxevanis@biol.uoa.gr (C.N.B.); 2 Biomedical Research Foundation, Academy of Athens, 11527 Athens, Greece; agapaki@bioacademy.gr (A.A.); palexakos@bioacademy.gr (P.A.); 3 Department of Pharmacognosy and Natural Products Chemistry, Faculty of Pharmacy, National and Kapodistrian University of Athens, Panepistimiopolis, Ilisia, 15774 Athens, Greece; gsarikaki@pharm.uoa.gr (G.S.); skaltsounis@pharm.uoa.gr (A.-L.S.); 4 Department of Plastic Surgery, Evaggelismos Hospital, 10676 Athens, Greece; drkyriakopoulos@gmail.com; 5 PharmaGnose S.A., 57 km National Road Athinon-Lamia, 32011 Inofyta, Greece; kmavreas@chem.uoa.gr; 6 Department of Pharmaceutical Chemistry, Faculty of Pharmacy, National and Kapodistrian University of Athens, Panepistimiopolis, Ilisia, 15771 Athens, Greece

**Keywords:** oleuropein, semi-synthetic analog, Ole-Oxy, anti-inflammatory activity, acute skin inflammation, acute colon inflammation

## Abstract

Inflammation is a key process in the pathophysiology of various diseases, with macrophages playing a central role in the inflammatory response. This study investigates the anti-inflammatory potential of a newly synthesized analog of oleuropein (OP), the major olive tree (*Olea europaea)* metabolite. This derivative of OP, named Ole-Oxy, was designed by introducing an oxygen atom between the aromatic ring and the aliphatic chain of OP, to enhance interaction with proteins and improve bioactivity. Ole-Oxy demonstrated notable anti-inflammatory effects in vitro, particularly in phorbol 12-myristate 13-acetate-differentiated THP-1 macrophages, where it markedly reduced interleukin-6, tumor necrosis factor-α, and reactive oxygen species (ROS) levels, surpassing the effects of OP. In vivo, Ole-Oxy was evaluated in mouse models of acute skin and colon inflammation, showing significant efficacy in C57BL/6J mice, likely due to their Th1-biased immune response. Our results suggest that Ole-Oxy modulates inflammation through ROS scavenging and differential macrophage activation, underscoring the need for further research to fully elucidate its mechanism of action and optimize its pharmacokinetic properties for future therapeutic applications.

## 1. Introduction

Inflammation and oxidative stress are fundamental processes implicated in the pathogenesis of various chronic diseases, including cardiovascular disorders [1], neurodegenerative diseases [2], and cancer [3]. Anti-inflammatory drugs are effective; however, short-term use may lead to metabolic acidosis, acute renal failure, and coma, while long-term use is linked to side effects such as stomach bleeding, increased blood pressure, elevated liver enzymes, and a heightened risk of stroke and heart attack [4]. Thus, the identification of less-toxic compounds with potent anti-inflammatory and antioxidant properties is intensively investigated [5].

Oleuropein (OP), a phenolic compound primarily found in olive leaves and fruit, has gained significant attention for its pharmacological potential [6]. OP has been extensively studied for its diverse pharmacological properties, particularly its anti-inflammatory, antioxidant, and anti-cancer effects. It has been shown to significantly inhibit pro-inflammatory mediators such as tumor necrosis factor (TNF)-α, interleukin (IL)-1β, IL-6, and IL-8, as well as oxidative stress markers like reactive oxygen species (ROS), both in vitro and in vivo [7]. However, despite its promising therapeutic potential, the low bioavailability and instability of OP have limited its clinical application. The acidic environment of the stomach degrades OP, and even the fraction that is absorbed is metabolized in the liver, thus reducing the circulating levels of intact OP [8]. To address these challenges, recent research focuses on the development of advanced formulations and structurally modified OP analogs, which aim to enhance its stability, absorption, and overall efficacy, paving the way for its effective use in medical treatments.

**Figure 1:**
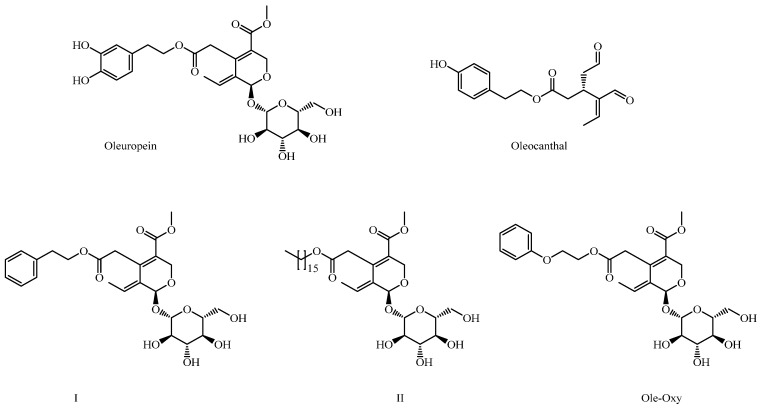
Structures of oleuropein (OP), oleocanthal and OP derivatives **I**, **II** and Ole-Oxy.

Our research team is intensively involved in the design, synthesis and assessment of structural analogs of key secondary metabolites of the olive tree (*Olea europaea*), including OP and oleocanthal (OC). In this context, we have synthesized a series of structural analogs of OP, in which the hydroxytyrosol moiety has been replaced with various other aromatic or aliphatic substituents. These derivatives have demonstrated significant anticancer activity and exhibit improved solubility and stability [9]. Among them, derivatives **I** and **II** were the most active, bearing a phenylethyl group or a large aliphatic chain, respectively (Figure 1).

In this study, as a continuation of our previous work, we synthesized Ole-Oxy, a new analog of compound I (Figure 1), in which an oxygen atom has been incorporated between the aromatic ring and the aliphatic chain. The absence of the catechol moiety in Ole-Oxy minimizes instability associated with catechol oxidation, while the addition of an oxygen atom may improve the compound’s solubility and adjust the length of the side chain—factors we previously identified as critical for activity [9]. The new molecule was tested both in vitro and in vivo, and showed well improved anti-inflammatory and antioxidant activities compared to OP. The mechanism(s) underlying the effects of Ole-Oxy provide insights into the potential therapeutic applications of semi-synthetic OP analogs in the management of inflammatory and oxidative-stress-related disorders.

## 2. Materials and Methods

### 2.1. Materials and Instruments

All commercially available chemicals and solvents were purchased from Fluorochem (Glossop, UK) and used as received without any further purification. All nuclear magnetic resonance (NMR) spectra were recorded on a Bruker Avance III 600 spectrometer (Bruker BioSpin GmbH, Rheinstetten, Germany). ^1^H-NMR (600 MHz) and ^13^C{^1^H}NMR (151 MHz, recorded with complete proton decoupling) spectra were obtained with samples dissolved in D_2_O or MeOD with the residual solvent signals used as internal references. Chemical shifts (*δ*) are given in ppm to the nearest 0.01 (^1^H) or 0.1 ppm (^13^C). The coupling constants (*J*) are given in Hertz (Hz). Assignments of ^1^H and ^13^C-NMR signals were unambiguously achieved with the help of D/H exchange and 2D techniques, namely COSY, NOESY, HMQC, and HMBC experiments. Flash chromatography was performed on Merck silica gel (40–63 μm) with the indicated solvent system using gradients of increasing polarity in most cases (Merck, Darmstadt, Germany). The reactions were monitored by analytical thin-layer chromatography (Merck pre-coated silica gel 60 F254 thin layer chromatography (TLC) plates, 0.25 mm layer thickness). Compounds were visualized on TLC plates by both UV radiation (254 and 365 nm) and spraying with vanillin as a staining agent (followed by subsequent warming with a heat gun). Mass spectra were recorded on a hybrid LTQ™ Orbitrap Discovery XL instrument (Thermo Fisher Scientific, Bremen, Germany), coupled to an Accela HPLC system (Thermo Fisher Scientific) equipped with a binary pump, an autosampler, and the Xcalibur 2.1 software.

### 2.2. Chemistry

#### 2.2.1. Synthesis of Oleoside or (4S,5E,6S)-4-(Carboxymethyl)-5-ethylidene-6-[(2S,3R,4S,5S,6R)-3,4,5-trihydroxy-6-(hydroxymethyl)oxan-2-yl]oxy-4H-pyran-3-carboxylic Acid (1)

To a solution of OP (5.5 g, 10.2 mmol in 50 mL H_2_O), at room temperature (rt), a cold NaOH solution (2 g, 50 mmol in 60 mL of H_2_O) was added dropwise. The resulting mixture was stirred for 24 h at rt, acidified with 2 N HCl (pH ~4.5), concentrated under reduced pressure, and purified by Centrifugal Partition Chromatography (CPC) to afford 3.5 g (85%) of the title compound. The centrifugal system of solvents was EtOAc/2-propanol/EtOH/H_2_O/Acetic acid 8/2/1/10/0.5 (8.6 L), with a column capacity of 1 L, rapid rotation at 800 rpm, and a flow rate of 15 mL/min. ^1^H NMR (600 MHz, D_2_O) *δ* 7.68 (s, 1H, H-3), 6.25 (q, *J*_8,10_ = 7.09 Hz, 1H, H-8), 6.04 (s, 1H, H-1), 5.04 (d, *J*_1′,2′_ = 8.03 Hz, 1H, H-1′), 4.11 (dd, *J*_5,6b_ = 9.63, *J*_5,6a_ = 4.57 Hz, 1H, H-5), 4.01 (dd, *J*_6a′,6b′_ = 12.49, *J*_6a′,5′_ = 2.20 Hz, 1H, H-6a′), 3.83 (dd, *J*_6b′,6a′_ = 12.49, *J*_6b′,5′_ = 5.92 Hz, 1H, H-6b′), 3.65–3.62 (m, 1H, H-3′), 3.61 (ddd, *J*_5′,4′_ = 8.20, *J*_5′,6b′_ = 5.92, *J*_5′,6a′_ = 2.20 Hz 1H, H-5′), 3.54–3.52 (m, 1H, H-4′), 3.52–3.50 (m, 1H, H-2′), 2.91 (dd, *J*_6a,6b_ = 13.67, *J*_6a,5_ = 4.57 Hz, 1H, H-6a), 2.54 (dd, *J*_6b,6a_ = 13.67, *J*_6b,5_ = 9.63 Hz, 1H, H-6b), 1.84 (dd, *J*_10,8_ = 7.12, *J*_10,1_ = 1.30 Hz, 3H, H-10); ^13^C NMR (151 MHz, D_2_O) δ 176.47 (C-7), 170.50 (C11), 154.62 (C-3), 128.46 (C-9), 125.06 (C-8), 108.68 (C-4), 99.73 (C-1′), 94.96 (C-1), 76.42 (C5′), 75.74 (C-3′), 72.73 (C-2′), 69.55 (C-4′), 60.71 (C-6′), 40.31 (C-6), 30.70 (C-5), 12.88 (C-10); HRMS (ESI-) m/z 389.1091 (calcd for C_16_H_21_O_11_ 389.1089).

#### 2.2.2. Synthesis of Methyl (4S,5E,6S)-4-[2-[2-(Phenoxy)ethoxy]-2-oxoethyl]-5-ethylidene-6-[(2S,3R,4S,5S,6R)-3,4,5-trihydroxy-6-(hydroxymethyl)oxan-2-yl]oxy-4H-pyran-3-carboxylate (Ole-Oxy)

Acetic anhydride (3.3 mL, 34.99 mmol, 17.5 equiv) was added to a solution of oleoside (808 mg, 2 mmol, 1 equiv, **1**) in pyridine (1.56 mL, 19.99 mmol, 10 equiv) at 0 °C. The resulting mixture was stirred at rt for 2 h, under argon. After completion of the reaction, as indicated upon TLC monitoring, the mixture was concentrated under reduced pressure to achieve anhydride **2**. Without any further purification, crude **2** was dissolved in dry acetonitrile (5 mL) and phenoxyethanol (106 μL, 0.845 mmol, 1.1 equiv), triethylamine (285 µL, 2.05 mmol, 2 equiv), and DMAP (125 mg, 1.02 mmol, 1 equiv) were added. The resulting solution was stirred at rt for 3 h, acidified with 2 N HCl (pH~4–5), and vacuum-evaporated. The residue was dissolved in CH_2_Cl_2_, washed with water, dried (Na_2_SO_4_), and concentrated to dryness to achieve crude **3**. Without further purification, the residue was dissolved in dry CH_2_Cl_2_ (5 mL). To this solution, 2,4,6-trichlorobenzoyl chloride (469 μL, 3 mmol, 1.5 equiv) and Et_3_N (389 µL, 2.8 mmol, 1.4 equiv) were added at 0 °C, and the resulting mixture was stirred at rt for 2 h, under argon. After completion of the reaction, as indicated by TLC monitoring, a solution of methanol (1 mL), DMAP (1 equiv) in dry CH_2_Cl_2_ (1 mL) was added dropwise, and the mixture was stirred for 3 h under argon. After the reaction was complete the resulting solution was acidified with 2 N HCl (pH ~4−5), washed with H_2_O, dried (anhydrous Na_2_SO_4_), and concentrated to dryness. Without further purification, crude **4** was dissolved in ethanol (85% aqueous) (5 mL) and diethylamine (364 μL, 7.5 mmol, 6 equiv) was added. The resulting solution was stirred for 6 h at 44 °C, acidified with 9% HCl (pH ~4–5), and evaporated to dryness. The residue was dissolved in EtOAc, washed with water, dried (Na_2_SO_4_), and concentrated to dryness. The crude product was purified by flash chromatography (silica gel), using CH_2_Cl_2_/MeOH, 100 → 85:15 *v/v* as the eluent, to achieve the title Ole-Oxy (530 mg, 52%).

^1^H NMR (600 MHz, MeOD) δ 7.54 (s, 1H, H-3), 7.32–7.27 (t, *J* = 8 Hz, 2H, H-5′/H-7′), 6.96 (m, 3H, H-4′/H-6′/H-8′), 6.15–6.09 (q, *J*_8,10_ = 7.07 Hz, 1H, H-8), 5.97 (s, 1H, H-1), 4.81 (d, *J*_1′′,2′′_ = 7.90 Hz, 1H, H-1′′), 4.46–4.40 (m, 1H, H-1a′), 4.36 (m, 1H, H-1b′), 4.20 (t, *J*_2′,1a′,1b′_ = 4.91 Hz, 2H, H-2′), 4.04 (dd, *J*_5,6b_ = 9.31, *J*_5,6a_ = 4.16 Hz, 1H, H-5), 3.89 (dd, *J*_6a′′,6b′′_ = 12.30, *J*_6a′′,5′′_ = 1.66 Hz, 1H, H-6a′′), 3.72 (s, 3H, COOCH_3_), 3.66 (dd, *J*_6b′′,6a′′_ = 12.20, *J*_6b′′,5′′_ = 5.53 Hz, 1H, H-6b′′), 3.42 (m, 1H, H-3′′), 3.36–3.28 (m, 3H, H-2′′, H-4′′, H-5′′), 2.78 (dd, *J*_6a,6b_ = 14.40, *J*_6a,5_ = 4.11 Hz, 1H, H-6a), 2.56 (dd, *J*_6b,6a_ = 14.08, *J*_6b,5_ = 9.53 Hz, 1H, H-6b), 1.76 (dd, *J*_10,8_ = 7.10, *J*_10,1_ = 1.30 Hz, 3H, H-10); ^13^C NMR (151 MHz, MeOD) δ 171.67 (C-7), 167.28 (C-11), 158.63 (C-3′), 153.77 (C-3), 129.19 (C-9), 129.11 (C-5′/C-7′), 123.49 (C-8), 120.69 (C-6′), 114.31 (C-4′/C-8′), 107.95 (C-4), 99.45 (C-1′′), 93.78 (C-1), 77.03 (C-5′′), 76.55 C-3′′), 73.38 C-2′′), 70.11 (C-4′′), 65.56 (C-2′), 62.99 (C-1′), 61.36 (C-6′′), 50.48 (COOCH_3_), 39.69 (C-6), 30.41 (C-5), 12.21 (C-10); HRMS (ESI+) m/z 525.1972 (calcd for C C_25_H_33_O_12_ 525.1967).

### 2.3. Cells and Cell Cultures

The human monocytic THP-1 and the mouse macrophage RAW 264.7 cell lines were purchased from the American Type Culture Collection (ATCC). Human peripheral blood (hPB) monocytes were isolated from healthy donors using Ficoll-Histopaque (Lonza, Basel, Switzerland), as described [10]. Naïve peritoneal-cavity cells were isolated from healthy C57BL/6J mice (10–12 weeks old) via peritoneal lavage that detaches, among other cells, resident macrophages from the peritoneal cavity (mouse peritoneal macrophages, mPM) [11]. Spleens from the same mice were removed under aseptic conditions and, after washing and red blood cell lysis, splenic macrophage-like cells (mouse splenic macrophages, mSM) were isolated, as previously described [12]. The study protocols were approved by the Department of Agriculture and Veterinary Service of the Prefecture of Athens (approval number: 497344/01-06-2022). All cells were cultured at 37 °C, 5% CO_2_, in RPMI-1640 medium supplemented with 10% *v/v* fetal bovine serum (FBS), 10 mM Hepes, and 10^2^ U/mL penicillin/streptomycin (hereafter complete medium; all from Lonza).

THP-1 monocytes were differentiated into macrophage-like cells after treatment with 100 ng/mL phorbol 12-myristate 13-acetate (PMA; Merck) for 24 h [13]. RAW 264.7 cells do not require PMA to exhibit their macrophage phenotype [14]. Primary cells were stimulated after incubation of hPB monocytes with 100 ng/mL of recombinant human (ImmunoTools GmbH, Friesoythe, Germany) and of mPM and mSM with mouse (Thermo Fisher Scientific) granulocyte–macrophage colony-stimulating factor (GM-CSF), for 5 consecutive days [15].

### 2.4. Cell Viability Assay

The half-maximal inhibitory concentrations (IC_50_) of OP and the new analog Ole-Oxy were determined using the 3-[4,5-dimethylthiazol-2-yl]-2,5 diphenyl tetrazolium bromide (MTT) dye-reduction assay. For the initial screening, OP and Ole-Oxy were diluted in dimethyl sulfoxide (DMSO; stock concentration 5 mM) and further, serially diluted (50–0.08 µM) with complete medium in 96-well plates (Greiner Bio-One GmbH, Frickenhausen, Germany), in which 5 × 10^4^ THP-1 macrophage-like or RAW 264.7 cells, and 1 × 10^5^ hPB macrophages, mPM or mSM were added per well at a final volume of 200 µL/well. Cells were incubated for 24 h in the presence or absence of the inflammatory response inducer lipopolysaccharide (LPS; 0.5 μg/mL; Sigma-Aldrich, St. Louis, MO, USA) [16]. Four hours before the end of incubation, 100 μL MTT (1 mg/mL; Sigma-Aldrich) diluted in phosphate-buffered saline (PBS; Lonza) was added to each well. The optical density (OD) of the wells was determined at 545 nm, using a SpectraMax^®^ ABS Plus reader (Molecular Devices, San Jose, CA, USA). IC_50_ values were calculated based on the measured ODs using GraphPad Prism 8.01 (GraphPad Software, San Diego, CA, USA).

### 2.5. Assessment of Cytokine Levels

The secretion of IL-6 and TNF-α was quantified in culture supernatants of both human and murine macrophage and macrophage-like cells incubated with LPS and OP (250 μΜ) or Ole-Oxy (10 μΜ) for 24 h using human and mouse Cytometric Bead Array (CBA) kits (BD Biosciences, Heidelberg, Germany; 550484 and 560485, respectively). Both assays were performed as previously described by us [17].

### 2.6. Measurement of Reactive Oxygen Species (ROS)

Intracellular ROS levels were quantified using 5-(6)-chloromethyl-2′,7′-dichlorodihydrofluorescein diacetate (CM-H_2_DCFDA; Molecular Probes, Eugene, OR, USA) as a cellular oxidative stress indicator, as described [18]. Macrophage-like THP-1 or RAW 264.7 cells (10^6^/mL) were incubated in 6-well plates (Greiner) for 4 h at 37 °C with OP (200 μM), Ole-Oxy (10 μM) or in complete medium (negative control) in the presence of 10 μΜ of the bacterial tripeptide N-formyl-L-methionyl-L-leucyl-L-phenylalanine (fMLP; Sigma-Aldrich), which stimulates the production of ROS [19]. Analysis was performed by flow cytometry on a BD FACSCanto II (BD Biosciences), using FACSDiva™ version 9.0 software (BD Biosciences).

### 2.7. Mouse Models

Mice were bred and maintained at the animal facility of the Biomedical Research Foundation of the Academy of Athens (BRFAA) under conditions specified in Samara et al. [9]. The study protocol was approved by the Department of Agriculture and Veterinary Service of the Prefecture of Athens (approval number: 497344/01-06-2022). For the inflammation models used, power analysis was performed in advance, using the G*power software (version 3.1.9.7, Universität Kiel, Germany), to determine the minimum sample size needed for enough statistical power to detect an effect. Assuming a reduction in the overall PASI score of 15% (±0.3%) in the treated vs. the control group after the 3rd day of treatment, power analysis determined the sample size at *n* = 4 mice/group, at a remarkably high power of 0.95 (with the lowest acceptable value being 0.81).

### 2.8. Toxicity Studies of Ole-Oxy

C57BL/6J and BALB/c male mice were randomly divided into nine groups (*n* = 3/group). The experimental groups for both strains received intraperitoneally (i.p.) OP (0.6–4.8 mg/dose) or Ole-Oxy (1–200 μg/dose) dissolved in PBS (200 μL) for 4 consecutive days (a total of 4 doses). The control group received i.p. PBS for the same number of doses (Figure S1). After each dose administration, the animals were kept under close observation for 30 min and intermittently for the next 8 h for the entire duration of the experiment. Throughout this period, clinical observations were made for motility, mortality, behavioural, neurological, or other abnormalities, and the weight of the mice was measured daily until the last day of experimentation. The parameters observed were as follows: general activity, vocally frantic, irritability, touch response, tail grip response, urination, defecation, and death.

### 2.9. Skin Inflammation Model

C57BL/6J (*n* = 16) and BALB/c (*n* = 16) male mice, 10–12 weeks old, were used. For the induction of imiquimod (IMQ)-induced psoriasiform dermatitis (AIPD), 62.5 mg of Modiwart cream (Iasis Pharma, Kamatero, Greece) containing 5% IMQ was topically applied daily on a 2 × 3 cm shaved back skin area of C57BL/6J (*n* = 12) and BALB/c (*n* = 12) mice for 6 consecutive days. In the remaining 4 animals of each strain, Vaseline was topically applied as a control [20]. IMQ-treated animals were randomly assigned into 3 groups/strain (*n* = 4/group). On day 0, they were injected i.p. with either PBS (200 μL; control group), OP (600 μg), or Ole-Oxy (2 or 5 and 20 or 50 μg/dose/mouse diluted in 200 μL PBS for C57BL/6J and BALB/c, respectively) daily, for 4 consecutive days (Figure S1A). Animal weight was measured daily. The thickness of the skin was also recorded on a daily basis, by measuring the double-fold skin thickness with a digital caliper. During the same time period, the inflamed area was macroscopically evaluated for the presence of desquamation and erythema using the Psoriasis Area and Severity Index scoring system [21] (PASI score 0–4; Table S1).

### 2.10. Colon Inflammation Model

Colon inflammation was induced as described by Guazelli et al. [22], with some modifications, to increase the survival of mice. A 3-day fasting period with ad libidum access to water and Ringer’s solution (35% dextrose; Vioser, Greece) preceded the induction of acute colon inflammation in both C57BL/6J and BALB/c mice. On day 0, C57BL/6J (*n* = 12) and BALB/c (*n* = 12) mice, were anesthetized with 2–3% isoflurane/O_2_ (IsoFlo^®^, Abbott, USA), and were administered a single intrarectal infusion of 5% acetic acid (AA) solution in 200 μL 0.9% NaCl, using a 19 mm 26 G polyethylene cannulum (Medikit, Gurgaon, India). The animals were further randomly assigned into 3 groups/strain (*n* = 4/group). Experimental groups received OP (600 μg), whereas Ole-Oxy (2 or 5 and 20 or 50 μg/dose/mouse diluted in 200 μL PBS, for C57BL/6J and BALB/c, respectively) and one group of each strain received intrarectally 0.9% NaCl and then, i.p. PBS daily for 4 consecutive days (Figure S1B). Animal weight was monitored daily. Intestinal length was measured postmortem, while intestinal ulcers, hyperemia, and inflammation were macroscopically evaluated using the Gerald classification system score [23] (score 1–10; Table S3).

### 2.11. Tissue Collection and Histological Staining

Animals were euthanized by cervical dislocation. The inflamed areas (both from skin and colon) were excised and fixed in 10% buffered formalin overnight and then embedded in paraffin for histological analysis. Sectioning and staining were performed as previously described [24]. Scores were assessed as shown in Tables S2 and S4.

### 2.12. Statistical Analysis

The results are expressed as means ± standard deviation (SD), unless otherwise stated. GraphPad Prism version 8.01 was used for data analysis. Paired *t* tests were used for statistical analysis, where *p* values of less than 0.05 were considered statistically significant.

## 3. Results

### 3.1. Chemical Synthesis of Ole-Oxy

**Scheme 1:**
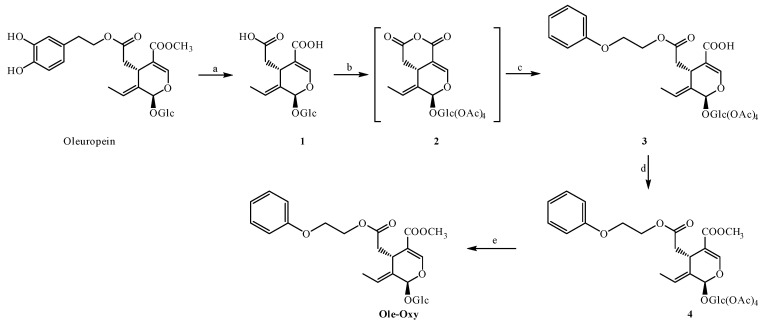
Reaction conditions: (a) NaOH, H_2_O, rt, 24 h; (b) Ac_2_O, py, rt, 24 h; (c) 2-phenoxyethanol, DMAP, Et_3_N, CH_2_Cl_2_, Ar, 0 °C to rt, 20 h; (d) (1) 2,4,6-trichlorobenzoyl chloride, Et_3_N, CH_2_Cl_2_, Ar, 0 °C (2) methanol; (e) Et_2_NH, MeOH, rt.

For the synthesis of Ole-Oxy, we utilized a protocol previously described from our research group [25], regarding the synthesis of OC analogs, with slight modifications also shown in Scheme 1. Briefly, OP extract, obtained from olive leaves, was hydrolyzed in one step to obtain the corresponding oleoside (**1**), which was then purified by fast centrifugal partition chromatography (FCPC). The treatment of oleoside (**1**) with acetic anhydride produced the mixed anhydride **2**, which upon reaction with 2-phenoxyethanol provided carboxylic acid **3**. Subsequently, carboxylic acid **3** was reacted with 2,4,6-trichlorobenzoyl chloride, and the resulting product was treated with methanol to achieve compound **4**. Finally, the deprotection of **4** with diethylamine provided the desired methyl ester Ole-Oxy. The total yield was 44%.

### 3.2. Ole-Oxy Reduces Inflammatory Responses in Cultured Human and Mouse Macrophages

**Figure 2:**
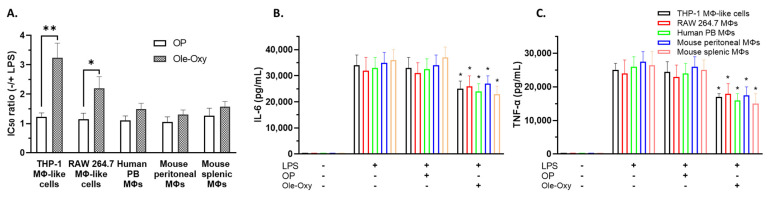
Ole-Oxy reduces lipopolysaccharide (LPS)-induced inflammation in human and mouse macrophages/macrophage-like cells. (**A**) Cells were stimulated or not with LPS (0.5 μg/mL; 24 h) before the addition of oleuropein (OP) or Ole-Oxy and the half-maximal inhibitory concentration (IC_50_) ratio (−/+ LPS) was determined. (**B**,**C**) Concentrations of IL-6 and TNF-α, measured in culture supernatants of the same cells. Means ± standard deviation (SD) values of three independent experiments are shown. MΦ, macrophage; PB, peripheral blood; +, presence; −, absence. *, *p* < 0.05; **, *p* < 0.01, compared to OP.

Macrophages constitute a cell population with a pivotal role in inflammation [26]. Thus, we initially tested Ole-Oxy in human and mouse macrophages, namely PMA-differentiated THP-1 macrophage-like cells, RAW 264.7 cells, GM-CSF-differentiated hPB macrophages, mPM, and mSM [27,28,29,30]. Using the MTT dye-reduction assay, the IC_50_ of Ole-Oxy in the absence or presence of LPS was determined in comparison to OP (Figure 2A).

As shown, in the presence of LPS, a three-fold lower concentration of Ole-Oxy was required to reduce the viability of LPS-stimulated macrophage-like THP-1 cells (*p* < 0.01), and a two-fold lower concentration to inhibit RAW 264.7 macrophages (*p* < 0.05), suggesting the cytoprotective activity of the analog. The corresponding ratio of OP was less prominent. Similarly, Ole-Oxy reduced the inflammatory responses in hPB macrophages and in mSM and mPM, when compared to OP, but to a lesser, statistically non-significant extent (Figure 2A). The supernatants of the aforementioned cell cultures contained less IL-6 and TNF-α when Ole-Oxy was used compared to OP (*p* < 0.05), further supporting the anti-inflammatory activity of Ole-Oxy (Figure 2B,C).

### 3.3. Ole-Oxy Shows Higher Antioxidant Activity Compared to OP

**Figure 3:**
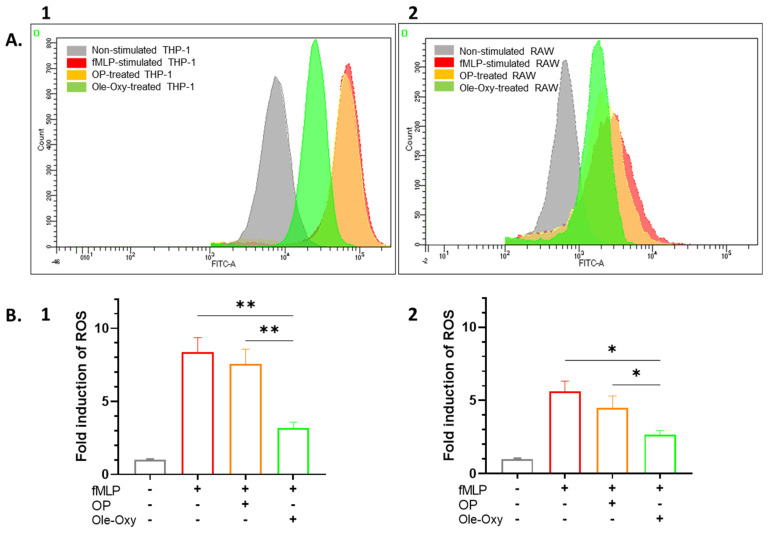
Antioxidant activity of Ole-Oxy against the bacterial tripeptide N-formyl-L-methionyl-L-leucyl-L-phenylalanine tripeptide (fMLP)-stimulated THP-1 (**1**) and RAW 264.7 cells (**2**). (**A**) Representative overlaid histograms of CM-H_2_DCFDA fluorescence in cells incubated only with fMLP (red; positive control), with oleuropein (OP, orange) or Ole-Oxy (green). THP-1 or RAW 264.7 cells incubated in complete medium (grey) were used as negative control. (**B**) Fold induction of reactive oxygen species (ROS) calculated as ratio of the mean fluorescence intensity (MFI) of CM-H_2_DCFDA in each culture condition (shown under x axis; +, presence; −, absence) over MFI of the negative control (grey bar) for THP-1 (**1**) and RAW 264.7 cells (**2**). Means ± standard deviation (SD) values of three independent experiments are shown. *, *p* < 0.05, **, *p* < 0.01.

The antioxidant activity of Ole-Oxy was tested against human THP-1 and mouse RAW 264.7 cells incubated with the bacterial tripeptide fMLP (5 µM; 4 h), and further stained with CM-H_2_DCFDA which diffuses passively into cells, followed by esterase-cleavage and oxidization by ROS, generating a fluorescent product. In the presence of Ole-Oxy, ROS production was markedly reduced compared to cells incubated with OP (Figure 3A). Comparing the mean fluorescence intensity (MFI) values of the dye in the different conditions, a statistically significant two-fold reduction in ROS was observed in THP-1 cells incubated with Ole-Oxy compared to control and OP-treated cells (*p* < 0.01; Figure 3B1), with a similar reduction (two-fold) seen for RAW 264.7 cells (*p* < 0.05; Figure 3B2).

### 3.4. Toxicity Assessment of Ole-Oxy

To select the appropriate doses for the acute skin and colon inflammation models, C57BL/6J (*n* = 3) and BALB/c (*n* = 3) mice were injected i.p. with increasing doses of OP (0.6–4.8 mg/dose) and Ole-Oxy (1–200 μg/dose) for a total of 4 doses. The animals were monitored for four days. The survival curves showed that C57BL/6J mice died at doses equal to or higher than 10 μg/dose of Ole-Oxy, while BALB/c mice died at doses over 100 μg/dose of Ole-Oxy (Figure S2). Based on these data, the selected doses of Ole-Oxy administration were 2 and 5 μg/dose for C57BL/6J mice, and 20 and 50 μg/dose for BALB/c mice. OP did not show any toxicity even at the highest dose of 4.8 mg, and the lower dose tested (0.6 mg) was selected for further experiments [9].

### 3.5. Ole-Oxy Reduces Imiquimod-Induced Acute Skin Inflammation

**Figure 4:**
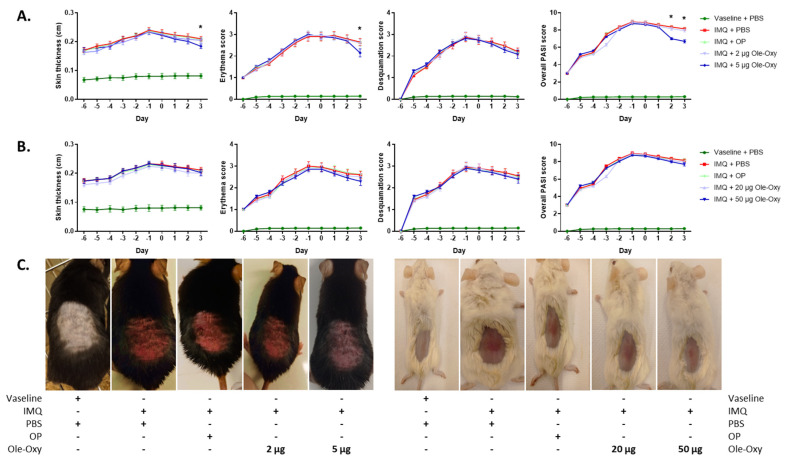
Effect of Ole-Oxy and oleuropein (OP) in imiquimod (IMQ)-induced skin inflammatory lesions in vivo. IMQ was applied daily for 6 consecutive days (−6 to −1) on the skin of (**A**) C57BL/6J and (**B**) BALB/c mice to induce a local inflammatory response. OP or Ole-Oxy were administered once daily intraperitoneally (i.p.) for 4 consecutive days (0 to +3). Control mice received PBS. Epidermal thickness (1st column) recorded using a digital caliper, erythema and skin desquamation (2nd and 3rd columns) were evaluated daily. The cumulative score (PASI) is presented in the last column. Pooled data from *n* = 4 mice/group are shown. (**C**) Representative photographs of C57BL/6J (left) and BALB/c mice (right) showing the extent of inflammation on day +3. *, *p* < 0.05, when comparing the 5 μg Ole-Oxy-treated group with the OP-treated group.

**Figure 5:**
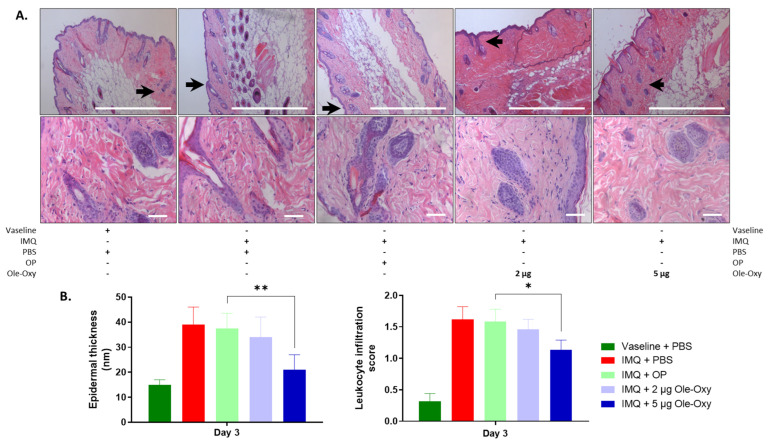
Effects of Ole-Oxy and oleuropein (OP) administration on epidermal thickness and inflamed tissue infiltration by leukocytes in C57BL/6J mice. (**A**) Representative photographs of histological sections of inflamed skin of C57BL/6J mice stained with haematoxylin/eosin (H/E), at 10× (top) and 40× (bottom) magnification. Scale bar, 100 μm. Arrows in 10× show areas enlarged at 40× magnification. (**B**) Bar charts summarizing the histopathological score of epidermal thickness (left) and leukocyte infiltration (right) based on pooled data from *n* = 4 mice/group. IMQ, imiquimod. *, *p* < 0.05; **, *p* < 0.01, compared with the OP-treated group.

The IMQ-induced acute skin inflammation in both C57BL/6J and BALB/c strains was followed both macroscopically by monitoring the evolution of erythema, desquamation, and skin thickness daily (Figure 4), and microscopically via histological analysis after the animals were sacrificed (Figure 5 and Figure S3).

In more detail, the severity of skin inflammation was macroscopically evaluated using a scoring system that is based on the clinical Psoriasis Area and Severity Index (PASI; Table S1). Erythema, desquamation, and skin thickness appeared to increase uniformly throughout the 6 days of IMQ administration in both animal strains (Figure 4), as a result of the progress of tissue inflammation.

In C57BL/6J mice, the measured double-fold skin thickness and erythema score were significantly reduced on day 3 in the group receiving 5 μg/dose of Ole-Oxy, when compared with the group receiving OP. Skin desquamation was unaffected between the experimental groups. The resulting cumulative PASI score (skin thickness, erythema and desquamation) was statistically significant during the last two days of 5 μg/dose Ole-Oxy administration in comparison to the OP-treated group. No other comparison between groups revealed statistical significance (Figure 4A). In BALB/c mice, the administration of Ole-Oxy showed no differences in any of the features investigated compared to control (Figure 4B).

Histologically, a variation of a semi-quantitative scoring system was used [31] (Table S2). Leukocyte infiltration and epidermal thickness were assessed across five fields of inflamed tissue per slide in C57BL/6J and BALB/c mice. At 10× magnification, modifications in the tissue architecture were depicted in all IMQ-administered groups in both animal strains, verifying the setting of inflammation. Specifically, in C57BL/6J mice (Figure 5A), epidermal thickness decreased statistically significantly in the group that received 5 μg/dose of Ole-Oxy, when compared with the OP-treated group. At 40× magnification, a statistically significant decrease in leukocyte infiltration at the same dosage was observed (Figure 5B). The lower Ole-Oxy dose (2 μg/dose) did not show any statistically significant differences in the criteria used when compared with the OP-treated group. In BALB/c mice, neither epidermal thickness nor leukocyte infiltration revealed any statistically significant differences between the Ole-Oxy- and OP-treated groups (Figure S3).

### 3.6. Ole-Oxy Reduces Acetic Acid-Induced Acute Colon Inflammation

**Figure 6:**
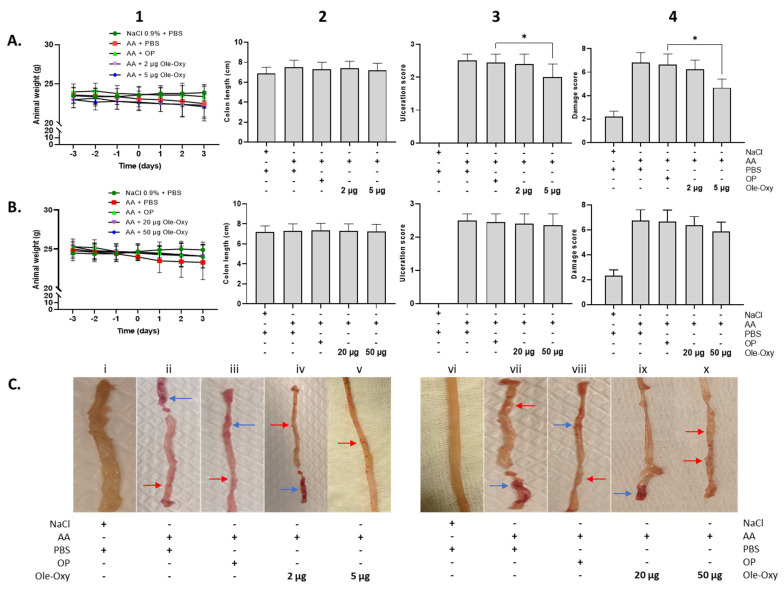
Effect of Ole-Oxy and oleuropein (OP) administration in acetic acid (AA)-induced colon inflammation in vivo. AA was administered intrarectally on day 0 to induce local inflammation in C57BL/6J (**A**) and BALB/c mice (**B**), after a 3-day fasting period. Ole-Oxy and OP were administered i.p. once daily for 4 consecutive days (0 to 3). Control mice received PBS. Animal weight (**1**) was recorded daily. The length of the colon (**2**), ulceration (**3**) and damage (**4**) scores were recorded postmortem (day 3). Pooled data from *n* = 4 mice/group are shown. (**C**) Representative pictures of colon preparations from both C57BL/6J (left; i–v) and BALB/c mice (right; vi–x) are shown. Red arrow—linear ulcer without significant inflammation; blue arrow—linear ulcer with inflammation at one focus. *, *p* < 0.05, compared with the OP-treated group.

**Figure 7:**
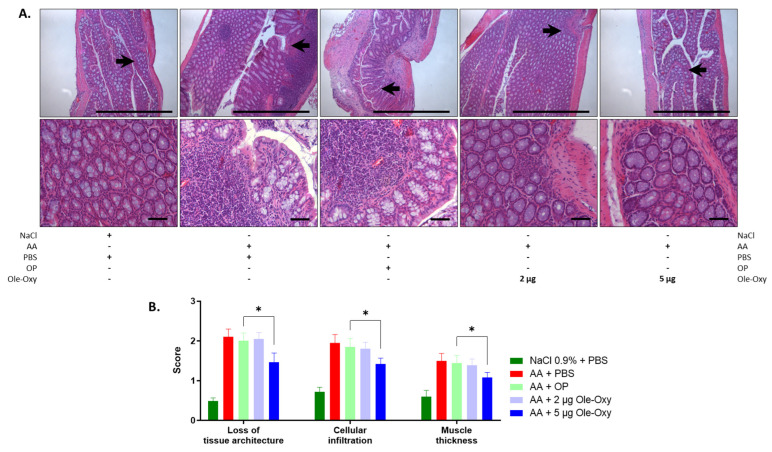
Effect of Ole-Oxy and OP on AA-induced colon inflammatory lesions in C57BL/6J mice. (**A**) Representative photographs of H/E-stained histological sections of inflamed tissue at 10× (top) and 40× (bottom) magnification. Scale bar, 100 μm. Arrows in 10× show areas enlarged at 40× magnification. (**B**) Bar chart summarizing the histopathological scores of the loss of tissue architecture, leukocyte infiltration and muscle thickness based on pooled data from *n* = 4 mice/group. *, *p* < 0.05, compared with the OP-treated group.

The progress of the induced acute colon inflammation in both animal strains (C57BL/6J and BALB/c) was evaluated both macroscopically (Figure 6) and microscopically (Figure 7 and Figure S4).

Macroscopically, animal weight, which directly reflects the well-being of the intestine (as part of the scoring system), did not show any significant variation among groups in either C57BL/6J or BALB/c mice (Figure 6(A1,B1)). Similarly, the length of the intestine, prepared from rectum to the cecal opening, was not significantly affected (Figure 6(A2,B2)). The presence and extent of intestinal ulcers decreased significantly only in C57BL/6J animals that received 5 μg/dose of Ole-Oxy, which showed linear ulcers without significant inflammation (Figure 6(A3,Cv)). On the contrary, the OP-treated group showed linear ulcers with inflammatory lesions at minimum one focus (Figure 6(A3,Ciii)). This affected the cumulative macroscopic damage score (Figure 6(A4), Table S3), that was also significantly reduced only in the 5 μg/dose of the Ole-Oxy-treated group (Figure 6(A4)). In BALB/c mice, no statistically significant changes between treated and control groups were observed in ulceration and overall damage scores (Figure 6(B3,B4)).

For the histopathological assessment, the scoring system described by Appleyard et al. [31] was used, with modifications. The features evaluated in the histological preparations were as follows: (a) loss of mucosal architecture, (b) leukocyte infiltration, (c) muscle wall thickness, (d) crypt abscess formation, and (e) goblet cell depletion (Table S4) in five fields per haematoxylin/eosin (H/E)-stained histological specimen. H/E staining revealed an increase in all parameters in all groups administered AA for the induction of colon inflammation.

In C57BL/6J mice (Figure 7A), the disruption of mucosal architecture and muscle wall thickness in the 5 μg/dose of the Ole-Oxy-treated group were significantly reduced when compared with the OP-treated group (*p* < 0.05) (Figure 7B). The 2 µg/dose of the Ole-Oxy-treated group showed no statistically significant decrease in any of the parameters evaluated. Leukocyte infiltration also increased significantly in the 5 μg/dose, but not in the 2 μg/dose of the Ole-Oxy groups when compared to the groups receiving OP. In BALB/c mice, no statistically significant differences were seen in any of the above-mentioned features assessed among experimental groups (Figure S4).

## 4. Discussion

Inflammation plays a well-established role in the pathophysiology of various diseases, including neurodegenerative disorders, cardiovascular conditions, aging, and cancer [32]. As a first-line defence mechanism, inflammation is driven by innate immune cells, with macrophages playing a central role. During infection, macrophages are typically activated by pathogen-associated stimuli, such as LPS, triggering the production of ROS and the release of pro-inflammatory cytokines. ROS production contributes to oxidative stress and tissue damage, while pro-inflammatory cytokines recruit additional immune cells and amplify the inflammatory signals [33].

We have previously designed, synthesized, and assessed a series of structural analogs of key secondary metabolites from the olive tree (*Olea europaea*), particularly focusing on derivatives of OP and OC, which demonstrated highly promising anticancer activity [9]. We showed that substituting the hydroxytyrosol moiety in OP with other aromatic (derivative **I** in Figure 1) or aliphatic (derivative **II** in Figure 1) groups improved the anticancer properties of the compounds, highlighting the importance of these structural modifications. Based on the activity of derivative **I**, we proceeded to design Ole-Oxy (Scheme 1). The insertion of an oxygen atom between the aromatic ring and the aliphatic chain was a strategic modification, as it could potentially enhance the compound’s anticancer activity by increasing its hydrogen-bonding capacity, likely strengthening interactions with proteins and improving affinity and specificity [34,35,36]. However, when tested against a series of human cancer cell lines, Ole-Oxy was completely inactive (IC_50_ > 50 μM; unpublished data). Considering that the oxygen atom can influence the electron distribution in the molecule, potentially enhancing its ability to scavenge free radicals and inhibit oxidative stress, both often associated with inflammation [37], we assessed the anti-inflammatory activity of Ole-Oxy.

The anti-inflammatory profile of OP and the newly synthesized Ole-Oxy was initially studied in vitro, using several cell-based models. To mimic the inflammatory response, macrophage-like THP-1 and RAW 264.7 cells, hPB macrophages, mPM and mSM were stimulated with LPS. Indeed, in the presence of LPS, incubation with Ole-Oxy significantly inhibited the activation of PMA-differentiated THP-1 macrophage-like cells and RAW 264.7 cells. LPS-induced activation of GM-CSF-differentiated macrophages (i.e., hPB macrophages, mSM, mPM) was also affected by Ole-Oxy, although not statistically significantly. The differential effects of Ole-Oxy in various macrophage types may be attributed to several factors, including differences in cell origin, differentiation pathways, and the LPS-activated signaling mechanisms. Macrophages derived from different sources can exhibit varying activation states and receptor expression profiles that affect LPS recognition and subsequent signaling [38]. Moreover, the differences in the cytokine milieu during differentiation (GM-CSF vs. PMA) can lead to variations in the functional phenotype and response to anti-inflammatory compounds [39]. In addition, the intracellular signaling pathways activated by LPS in different macrophage types can vary [40], potentially affecting the efficacy of anti-inflammatory agents like Ole-Oxy. For instance, LPS-induced activation of the nuclear factor (NF)-κB and mitogen-activated protein kinase (MAPK) pathways might differ in primary macrophages compared to the THP-1 and RAW 264.7 cell lines, leading to variable modulation by Ole-Oxy. Overall, the observed significant inhibition of PMA-differentiated THP-1 cells and RAW 264.7 cells compared to the less pronounced effects on GM-CSF-differentiated primary macrophages (hPB macrophages, mSM, mPM) by Ole-Oxy may be due to a combination of these factors.

Using PMA-differentiated THP-1 cells that showed a stronger induction of pro-inflammatory cytokines compared to parental cells [41], and RAW 264.7 cells that also displayed similar behavior, the cytokine content of their supernatants was quantified after exposure to OP and Ole-Oxy. The levels of IL-6 and TNF-α subsided significantly in the presence of Ole-Oxy compared with OP, which further corroborates its improved anti-inflammatory activity. IL-6 and TNF-α are key pro-inflammatory cytokines involved in the early phase of inflammation. IL-6 promotes acute inflammatory responses by stimulating the production of acute-phase proteins in the liver and by enhancing the differentiation of T cells [42], while TNF-α promotes the recruitment of immune cells to the site of infection or injury and stimulates the production of other pro-inflammatory cytokines (like IL-6) [43]. In our case, this IL-6 and TNF-α reduction in the presence of Ole-Oxy suggests that the compound effectively affects their production. Although not studied herein, this could be due to inhibition of the NF-κB signaling pathway, which is primarily responsible for the transcription of these pro-inflammatory cytokines [44]. Furthermore, the marked reduction in ROS production of PMA-differentiated THP-1 cells and RAW 264.7 cells after Ole-Oxy treatment confirms the enhanced antioxidant potential of the analog in comparison to OP. ROS are known to activate pro-inflammatory pathways, including NF-κB [44]. By reducing ROS levels more effectively than OP, Ole-Oxy might prevent the activation of these pathways, thereby leading to a greater reduction in pro-inflammatory cytokines.

In vivo, Ole-Oxy was evaluated in two models of inflammation established in two different mouse strains, displaying variable efficacy. In C57BL/6J mice, Ole-Oxy showed significant anti-inflammatory effects in both IMQ-induced skin and AA-induced colon inflammation. Macroscopically, the reduced inflammation scores observed, including erythema, desquamation, skin thickness and overall PASI scores in the skin inflammation model, and the corresponding scores in the colon inflammation model, namely colon length, ulceration, and collective damage, highlight the effectiveness of Ole-Oxy. Microscopically, the reduction in histological markers of inflammation, such as epidermal thickness and leukocyte infiltration in the inflamed skin, and loss of mucosal architecture, leukocyte infiltration and muscle wall thickness in the inflamed colon, further support the potential anti-inflammatory activity of Ole-Oxy. The overall decrease in inflammation scores, both macroscopic and histological, suggests that Ole-Oxy can modulate the inflammatory response effectively.

The differential efficacy of Ole-Oxy in C57BL/6J and BALB/c mice can likely be linked to several factors. Firstly, C57BL/6J and BALB/c mice possess distinct genetic backgrounds that result in differences in their immune responses, including innate immunity [45]. C57BL/6J mice are generally considered to have a Th1-biased immune response, characterized by increased production of pro-inflammatory cytokines such as interferon (IFN)-γ, while BALB/c mice have intrinsically a Th2-biased immune response, associated with higher production of Th2-type cytokines like IL-4, IL-5, and IL-13 [46]. This could explain why Ole-Oxy, which preferentially modulates Th1-driven inflammation, showed greater efficacy in C57BL/6J mice, markedly at a 10-fold lower dose (5 vs. 50 μg for C57BL/6J and BALB/c, respectively). Moreover, the variable effectiveness observed could be due to the presence of differentially polarized M1 in C57BL/6J and M2 in BALB/c macrophages, as has been previously reported for lung macrophages [47]. Secondly, the metabolic rate and drug absorption, distribution, metabolism, and excretion (ADME) may also vary between mouse strains [48]. C57BL/6J and BALB/c mice may metabolize Ole-Oxy differently, leading to variations in bioavailability and effective drug concentration at the site of inflammation. Lastly, the specific models of inflammation used (IMQ-induced skin and AA-induced colon inflammation) could interact differently with the genetic and immunological background of the mouse strains. C57BL/6J mice may show more pronounced symptoms or may respond differently to the inflammatory challenge, making it easier to monitor therapeutic effects. BALB/c mice, on the other hand, may have lower baseline levels of pro-inflammatory cytokines in response to the same inflammatory stimuli, which could influence the anti-inflammatory efficacy of the treatment [45,49].

While this study provides promising data on the efficacy of Ole-Oxy, there are limitations that warrant consideration. The in vitro and in vivo models used, though informative, may not fully simulate the complexity of human inflammatory diseases. Future studies should focus on elucidating the detailed mechanisms of action of Ole-Oxy optimizing its pharmacokinetic properties across several models of inflammation, including chronic inflammation, and related diseases which are crucial to fully understanding its therapeutic potential. This could be performed by exploring possible interference in specific intracellular signaling pathways and/or enzymatic interactions, e.g., the NF-κB pathway, a critical regulator of inflammatory responses [44]; the MAPK signaling pathway (ERK, JNK, and p38 MAPK) [50]; the Nrf2 pathway, essential for cell defence against oxidative stress [51]; and the arachidonic acid signaling pathway with its associated enzymes, such as lipoxygenase (LOX) and NADPH oxidase (NOX) [52]. Understanding such interactions will provide a more comprehensive view of the therapeutic potential of this new OP analog and could guide the development of optimized anti-inflammatory agents.

## 5. Conclusions

Based on previous structure–activity relationship (SARs) studies performed on OP analogs, a new molecule with significant anti-inflammatory activity was designed and synthesized. In vitro, Ole-Oxy mitigated LPS-induced inflammation more effectively than OP. When used in C57BL/6J models of acute IMQ-induced skin and AA-induced colon inflammation, Ole-Oxy significantly decreased all related inflammation scores as observed both macroscopically and histologically.

The improved properties of this new OP analog enrich our understanding of how SARs govern its anti-inflammatory and antioxidant effects, setting the basis for the development of novel therapeutic agents. While our findings are promising, further studies are necessary to elucidate the mechanisms of action and to evaluate the compound’s long-term efficacy and safety in additional preclinical models. Nevertheless, Ole-Oxy could serve as a lead compound for the development of new therapeutic agents targeting inflammatory and oxidative-stress-related diseases.

## Data Availability

The original contributions presented in the study are included in the article/Supplementary Material, further inquiries can be directed to the corresponding authors.

## Supplementary Materials

The following supporting information can be downloaded at: https://www.mdpi.com/article/10.3390/antiox13111422/s1, Figure S1: Protocols for animal models of IMQ-induced acute skin inflammation and AA-induced acute colon inflammation; Figure S2: Toxicity studies of Ole-Oxy in C57BL/6J and BALB/c mice; Figure S3: Effects of Ole-Oxy and OP on IMQ-induced skin lesions in BALB/c mice; Figure S4: Effects of Ole-Oxy and OP on AA-induced colon inflammatory lesions in BALB/c mice; Figure S5: ^1^H NMR of Ole-Oxy in MeOD; Figure S6: ^13^C NMR of Ole-Oxy in MeOD; Table S1: Macroscopic grading in the acute skin inflammation model; Table S2: Histopathological grading in the acute skin inflammation model; Table S3: Macroscopic grading in the acute colon inflammation model; Table S4: Histopathological grading in the acute colon inflammation model.
