# Supplementary material for: Ole-Oxy, a Semi-Synthetic Analog of Oleuropein, Ameliorates Acute Skin and Colon Inflammation in Mice
Source: Antioxidants (Basel). 2024 Nov 20;13(11):1422. doi: 10.3390/antiox13111422 (PMC11590887; doi:10.3390/antiox13111422)
Supplement: Supplementary file 1 [file antioxidants-13-01422-s001.zip › antioxidants-3281010-supplementary.docx]

**Supplementary Materials**

Ole-Oxy, a semi-synthetic analog of oleuropein ameliorates acute skin and colon inflammation in mice

Nikolaos V. Angelis^1^, Efthimios Paronis^1^, Georgia Sarikaki^2^, Antonios Kyriakopoulos^3^, Anna Agapaki^4^, Pigi-Maria Niotopoulou^1^, Christina Knai^1^, Pavlos Alexakos^4^, Odyssefs Liagkas^1^, Konstantinos F. Mavreas^5^, Constantin N. Baxevanis^1^, Alexios-Leandros Skaltsounis^2†^, Ourania E. Tsitsilonis^1†*^, Ioannis K. Kostakis^6†*^

^1^Flow Cytometry Unit, Section of Animal and Human Physiology, Department of Biology, National and Kapodistrian University of Athens, Panepistimiopolis, 15784, Ilisia, Athens, Greece

^2^Department of Pharmacognosy and Natural Products Chemistry, Faculty of Pharmacy, National and Kapodistrian University of Athens, Panepistimiopolis, 15774, Ilisia, Athens, Greece

^3^Department of Plastic Surgery, Evaggelismos Hospital, 10676, Athens, Greece

^4^Biomedical Research Foundation, Academy of Athens, 11527, Athens, Greece

^5^PharmaGnose S.A., 57 km National Road Athinon-Lamia, Inofyta, Greece

^6^Department of Pharmaceutical Chemistry, Faculty of Pharmacy, National and Kapodistrian University of Athens, Panepistimiopolis, 15771, Ilisia, Athens, Greece

†These authors contributed equally to this work

^*^Corresponding authors

Ioannis K. Kostakis, tel: +30 210 7274212; e-mail: [ikkostakis@pharm.uoa.gr](mailto:ikkostakis@pharm.uoa.gr)

Ourania E. Tsitsilonis, tel: +30 210 7274215; e-mail: [rtsitsil@biol.uoa.gr](mailto:rtsitsil@biol.uoa.gr)


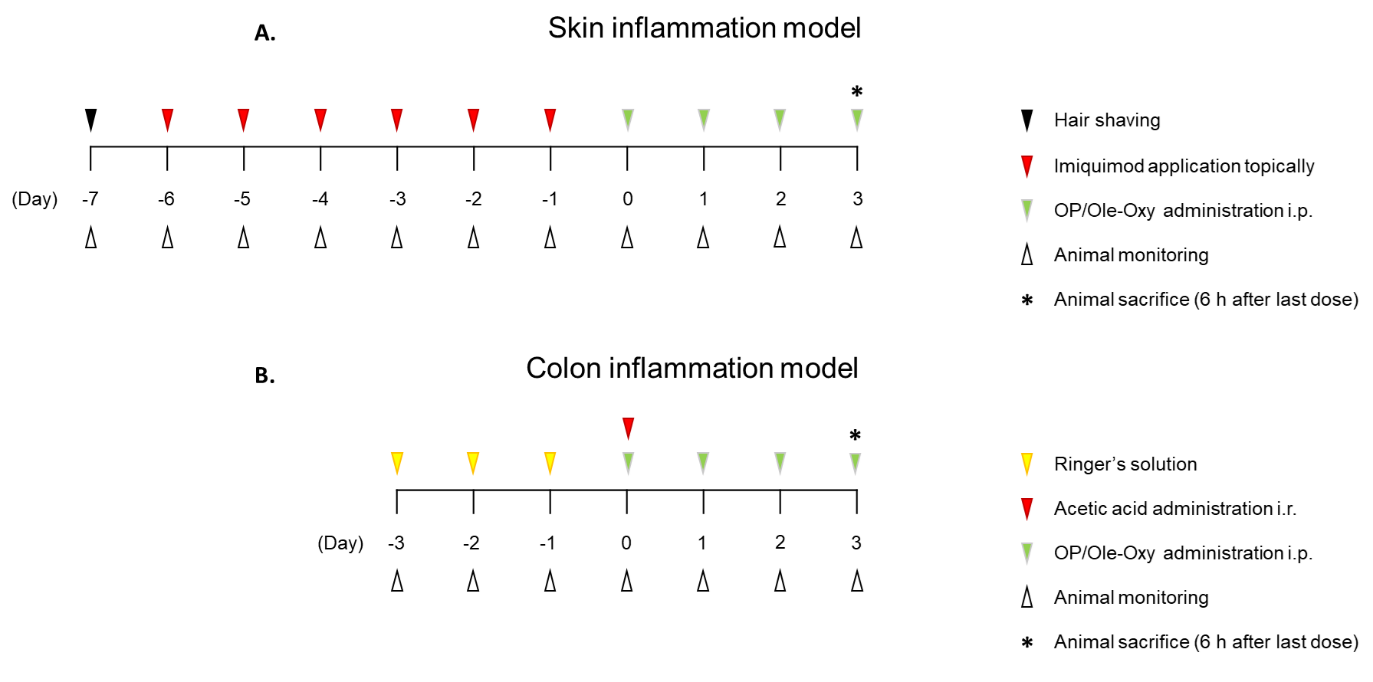


**Figure S1.** Protocols for animal models of imiquimod (IMQ)-induced acute skin inflammation and acetic acid (AA)-induced acute colon inflammation. Timeline for establishing acute skin (**A**) and colon (**B**) inflammation in both C57BL/6J and BALB/c mice, including the dosing schedule. (**A**) Hair shaving was performed on day -7, and IMQ (5%) was topically applied daily for 6 consecutive days (-6 to -1). Vaseline was used in the control group. On day 0, mice were injected i.p. with either PBS (200 μL; control group), oleuropein (OP) (600 μg) or Ole-Oxy (2 or 5 and 20 or 50 μg/dose/mouse diluted in 200 μL PBS for C57BL/6J and BALB/c, respectively) daily for 4 consecutive days (0 to 3). Animals were sacrificed 6 h after the last dose administration. (**B**) After a 3-day fasting period (water and Ringer’s solution, days -3 to -1), on day 0, C57BL/6J and BALB/c mice were administered a single intrarectal infusion of 5% AA solution in 200 μL 0.9% NaCl. Six h later, experimental groups received OP (600 μg) or Ole-Oxy (2 or 5 and 20 or 50 μg/dose/mouse diluted in 200 μL PBS, for C57BL/6J and BALB/c, respectively), for 4 consecutive days (0 to 3). Control groups received intrarectally 0.9% NaCl and i.p. PBS. Mice were euthanized 6 h after the last dose.


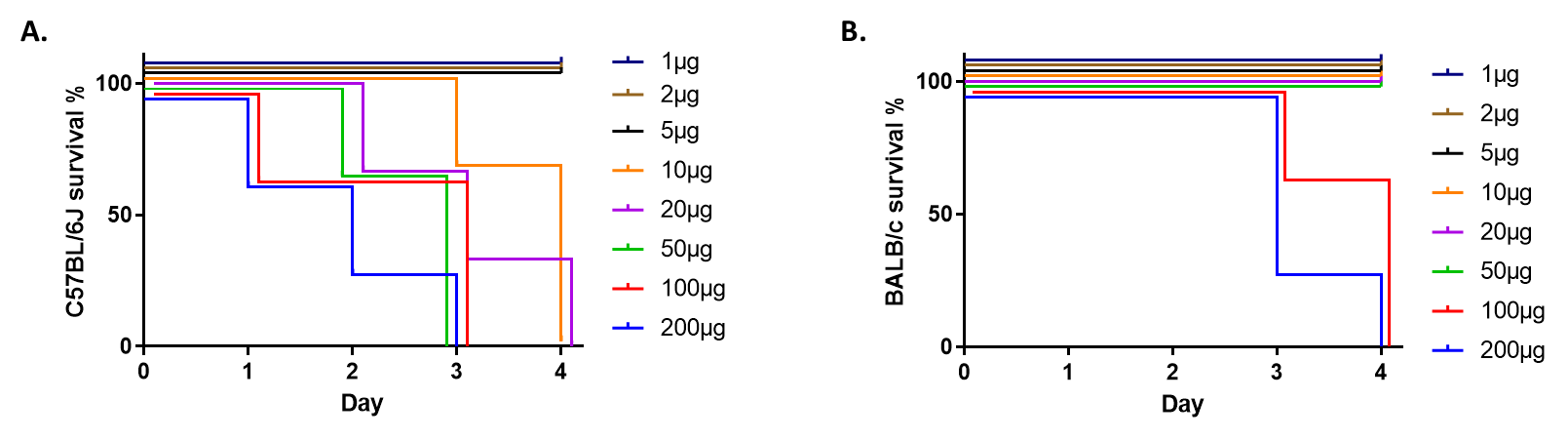


**Figure S2.** Toxicity studies of Ole-Oxy in C57BL/6J and BALB/c mice. Survival curves of healthy C57BL/6J (**A**) and BALB/c (**B**) mice receiving Ole-Oxy. Doses were administered i.p. daily for 4 consecutive days (0 to 3) and animals were monitored for an additional 24 h (day 4). Lethal dose of Ole-Oxy was estimated following *in vivo* exposure of healthy mice (n=3/group) to concentrations of Ole-Oxy ranging from 1-200 μg/dose. In C57BL/6J mice, Ole-Oxy was lethal at doses ≥10 μg, while the respective dose for BALB/c mice was ≥100 μg. (OP was administered daily at doses of 600 – 1,200 – 2,400 – 4,800 μg and showed no toxicity; data not shown).


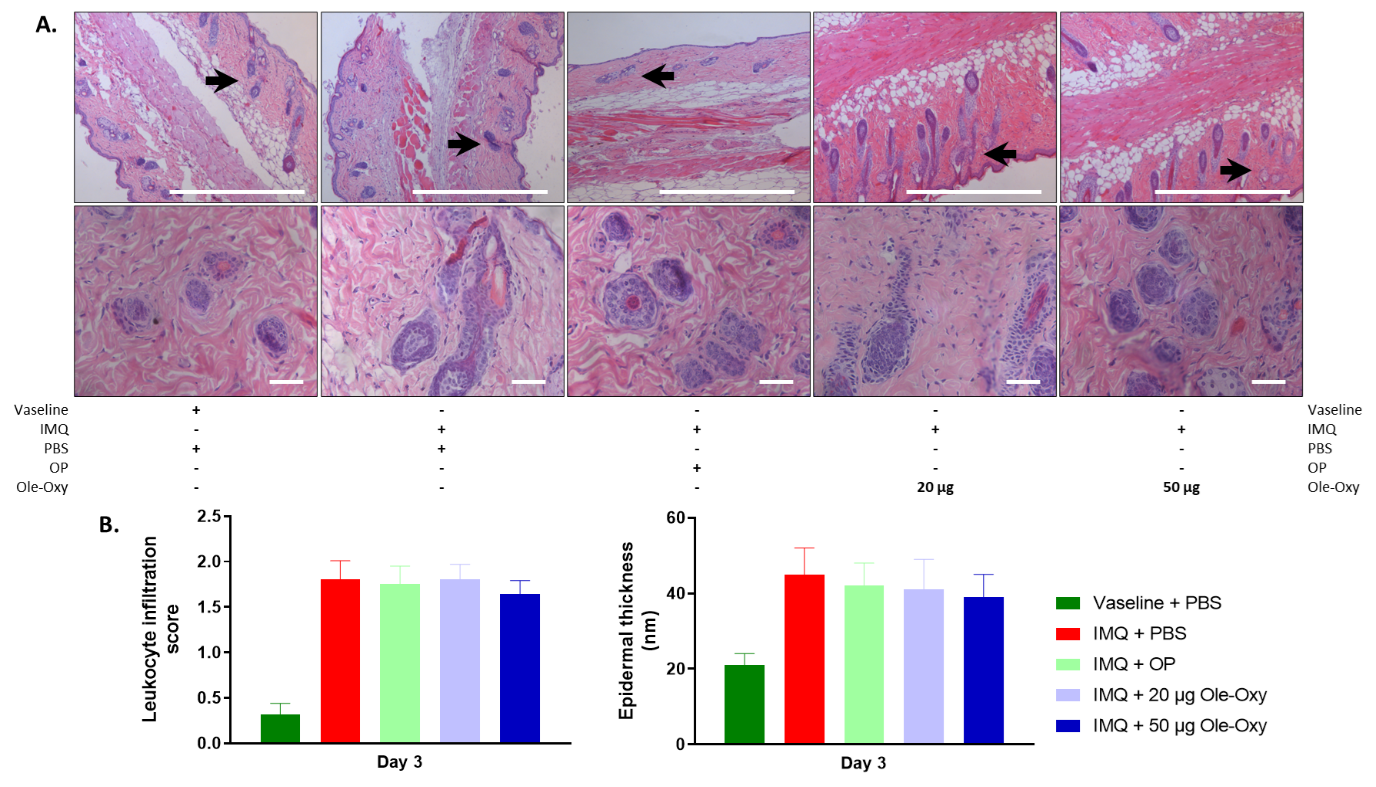


**Figure S3.** Effects of Ole-Oxy and OP on IMQ-induced skin lesions in BALB/c mice. (**A**) Representative photographs of H/E-stained histological sections of inflamed tissue of BALB/c mice at 10× (top) and 40× (bottom) magnification. Scale bar 100 μm. Arrows in 10× show areas enlarged at 40× magnification. (**B**) Bar chart summarizing the histopathological scores of leukocyte infiltration and loss of tissue architecture based on pooled data from 4 mice/group.


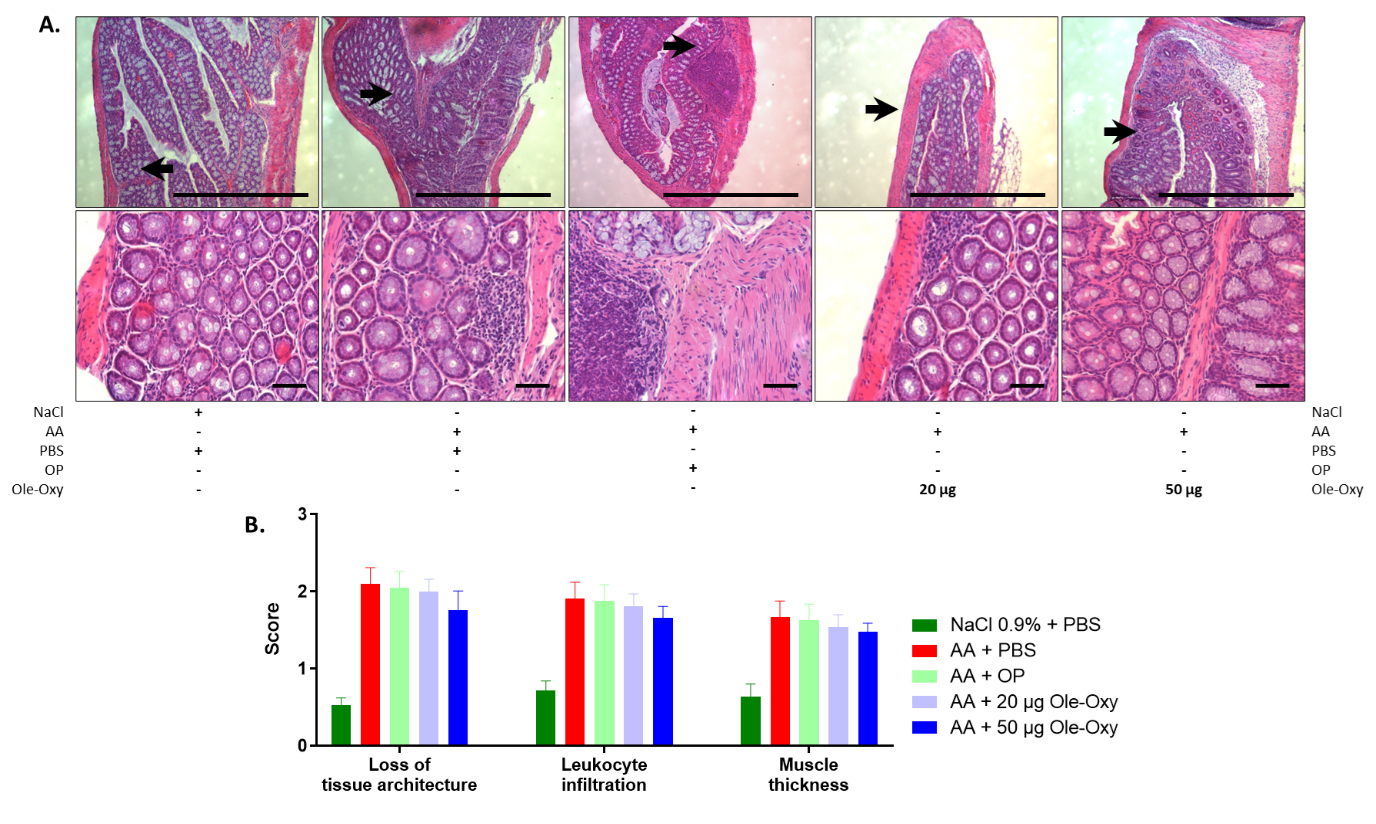


**Figure S4.** Effects of Ole-Oxy and OP on AA-induced colon inflammatory lesions in BALB/c mice. (**A**) Representative photographs of H/E-stained histological sections of inflamed tissue of BALB/c mice at 10× (top) and 40× (bottom) magnification. Scale bar 100 μm. Arrows in 10× show areas enlarged at 40× magnification. (**B**) Bar chart summarizing the histopathological scores of the loss of tissue architecture, leukocyte infiltration and muscle thickness based on pooled data from 4 mice/group.

**Figure S5.** ^1^H NMR of Ole-Oxy in MeOD.

**Figure S6.** ^13^C NMR of Ole-Oxy in MeOD.

**Table S1.** Macroscopic grading in the acute skin inflammation model.

| **Erythema**  **Desquamation**  **Skin Thickness** | **Score** | **Psoriasis Area and Severity Index (PASI)** |
| --- | --- | --- |
|  | 0 | None |
|  | 1 | Slight |
|  | 2 | Moderate |
|  | 3 | Marked |
|  | 4 | Very marked |

**Table S2.** Histopathological grading in the acute skin inflammation model.

| **Epidermal thickness**  **Leukocyte infiltration** | **Score** | **Histopathological grading** |
| --- | --- | --- |
|  | 0 | Normal |
|  | 1 | Low intensity |
|  | 2 | Intermediate intensity |
|  | 3 | High intensity |

**Table S3.** Macroscopic grading in the acute colon inflammation model.

| **Grade** | **Macroscopic grading** |
| --- | --- |
| 0 | Local hyperemia, no ulcers |
| 1 | Linear ulcers without significant inflammation |
| 2 | Linear ulcers, presence of inflammation in one focus |
| 3 | Two or more foci of linear ulcers and inflammation |
| 4 | One focus of inflammation >1 cm along the bowel |
| 5 | One focus of inflammation >2 cm along the bowel |
| 6-10 | Score increase by 1 for each additional cm |

**Table S4.** Histopathological grading in the acute colon inflammation model.

| **Mucosal architecture**  **Leukocyte infiltration**  **Muscle thickness** | | **Score** | | **Histopathological grading** |
| --- | --- | --- | --- | --- |
|  |  | 0 | | Normal |
|  |  | 1 | | Low intensity |
|  |  | 2 | | Intermediate intensity |
|  |  | 3 | | High intensity |
|  | |  | |  |
| **Crypt abscess formation**  **Goblet cell depletion** | **Score** | | **Histopathological grading** | |
|  | 0 | | Absent | |
|  | 1 | | Present | |
